# Phenotypic and genomic analyses of bacteriocin-producing probiotic *Enterococcus faecium* EFEL8600 isolated from Korean soy-meju

**DOI:** 10.3389/fmicb.2023.1237442

**Published:** 2023-09-04

**Authors:** Da Hye Kim, Seul-Ah Kim, Na Gyeong Jo, Jae-Han Bae, Minh Tri Nguyen, Yu Mi Jo, Nam Soo Han

**Affiliations:** Brain Korea 21 Center for Bio-Health Industry, Division of Animal, Horticultural, and Food Sciences, Chungbuk National University, Cheongju, Republic of Korea

**Keywords:** probiotic, lactic acid bacteria, soy-milk, bacteriocin, *Enterococcus faecium*, *Listeria* inhibition

## Abstract

*Enterococcus faecium* is a prevalent species found in fermented soybean products, known for its contributions to flavor development and inhibition of pathogenic microorganisms during fermentation. This study aims to provide comprehensive phenotypic and genomic evidence supporting the probiotic characteristics of *E. faecium* EFEL8600, a bacteriocin-producing strain isolated from Korean soy-meju. Phenotypic analysis revealed that EFEL8600 produced a peptide with inhibitory activity against *Listeria monocytogenes*, estimated to be 4.6 kDa, corresponding to the size of enterocins P or Q. Furthermore, EFEL8600 exhibited probiotic traits, such as resilience in gastrointestinal conditions, antioxidant and anti-inflammatory activities, and protection of the intestinal barrier. Safety assessments demonstrated no hemolytic and bile salt deconjugation activities. Genomic analysis revealed the presence of several genes associated with probiotic characteristics and bacteriocin production, while few deleterious genes with a low likelihood of expression or transferring were detected. Overall, this study highlights *E. faecium* EFEL8600 as a potent anti-listeria probiotic strain suitable for use as a starter culture in soymilk fermentation, providing potential health benefits to consumers.

## 1. Introduction

Soybean, a widely consumed legume, contains a diverse array of functional components, including isoflavones, soyasaponins, terpenes, and sterols, alongside anti-nutritional factors such as phytates and trypsin inhibitors (Licandro et al., 2020). The fermentation mediated by lactic acid bacteria (LAB) can effectively reduce phytates and trypsin inhibitors, and also facilitate the hydrolysis of tannic acid through their tannase activities leading to the generation of novel compounds that were not originally present in raw soybean (Jang et al., 2021). However, *Listeria monocytogenes*, a gram-positive foodborne pathogen, has been detected or presented a high risk of contamination in soy-based products, including soymilk (Haraguchi et al., 2019), soybean paste (Nam et al., 2012), and tempeh (Ashenafi, 1991). Consequently, the development of an effective fermentation process to ensure the safety of soy products has become an imperative task.

Bacteriocins are defined as a narrow range of antimicrobial peptides produced by major lineages of bacteria and some members of the Archaea (Bharti et al., 2015). Bacteriocin production can be beneficial to the host microbes by inhibiting or competing with other bacteria in the same environmental niche (Deegan et al., 2006). Many bacteriocins produced from lactic acid bacteria can be applied in the industry for food fermentation because they can suppress the growth of many problematic microorganisms in processed foods (Cotter et al., 2005). In the case of nisin, which is produced by *Lactococcus lactis,* has already been approved as a GRAS (generally regarded as safe) for food additive (Özel et al., 2018).

Enterococci are increasingly investigated as a potential probiotic candidate, and *Enterococcus faecium* is typically available as a probiotic on the market (Zommiti et al., 2018). The health-beneficial effects of *E. faecium* were reported as lowering cholesterol levels (Singhal et al., 2019), alleviating diarrhea (Torres-Henderson et al., 2017), and regulating the immune system (Franz et al., 2011). Notably, several studies reported that *Enterococcus* spp. can produce bacteriocins against foodborne pathogens such as *Listeria* spp. which belonging to various classes (Favaro et al., 2014). Especially, enterocin, which is representative bacteriocin produced by *Enterococcus*, is composed of small molecular weight peptides and has specific anti-microbial activity against gram-positive and putrefactive bacteria (Franz et al., 2011). Previously, several studies were reported on bacteriocin produced by *E. faecium*, including enterocin A, B, P, L50a, L50b, and Q (Valledor et al., 2022).

Meju, a solid-state soybean preculture consisting of a microbial consortium including bacteria and fungi, plays a crucial role as both an inoculum and a substrate in the fermentation process of traditional Korean soy-based foods such as doenjang (soybean paste) and ganjang (soybean sauce) (Jung et al., 2014). Throughout the fermentation process, the diverse enzymes secreted by the microbial community present in meju facilitate the hydrolysis of soybean polypeptides, carbohydrates, lipids, and isoflavone glycosides, resulting in the production of amino acids, sugars, organic acids, aglycones, and flavor compounds (Han et al., 2023). Notably, among the bacterial species inhabiting meju, *Enterococcus faecium* has been identified as the predominant microorganism due to its fast growth rate in soybean cultures and its ability to contribute to the development of desirable flavors in fermented foods (Jeong et al., 2019; Kumari et al., 2022).

Within the same context, we isolated various *E. faecium* strains from soy-meju and tested their anti-microbial and probiotic activities. Following comprehensive analysis, we identified *E. faecium* EFEL8600 as a superior strain. This study presents an in-depth investigation into the phenotypic characteristics of *E. faecium* EFEL8600, encompassing microbial and biochemical traits, probiotic properties (including safety, gastrointestinal stability, and health-promoting effects), and its capacity for bacteriocin production. Additionally, we provide insights into the genetic characteristics of EFEL8600, focusing on the presence of genes associated with safety, probiotic properties, and bacteriocin production. By elucidating these aspects, our study contributes to the understanding of *E. faecium* EFEL8600 as a promising candidate for potential applications in soybean fermentation.

## 2. Materials and methods

### 2.1. Bacterial isolation and culture conditions

*E. faecium* EFEL8600 was isolated from soy-meju and cultured in MRS medium (BD Difco^™^, Sparks, MD, United States) supplemented with 0.002% of bromophenol blue at a temperature of 37°C under aerobic condition without shaking. *Listeria monocytogenes* KCTC 3569 used as pathogenic bacteria were cultivated in BHI medium (BD Difco^™^) at a temperature of 37°C under anaerobic condition. The specific cultural conditions and growth media employed for other microorganisms utilized in this study are detailed in Table 1. *Lacticaseibacillus rhamnosus* GG (LGG) and *Lactiplantibacillus plantarum* WCFS1 (WCFS1) were selected as the reference probiotic strains for comparative analysis.

**Table 1:** Microorganism used in this study.

Species	Collections	Culture condition
*Enterococcus faecium* EFEL8600	KCTC 14743BP	37°C, MRS
*Enterococcus faecium*	DSM 20477	37°C, MRS
*Lacticaseibacillus rhamnosus* GG	KCTC 5033	37°C, MRS
*Lactiplantibacillus plantarum* WCFS1	ATCC BAA-793	37°C, MRS
*Listeria monocytogenes*	KCTC 3569	37°C, BHI
*Limosilactobacillus reuteri*	ATCC 23272	37°C, MRS
*Enterococcus faecalis*	KCCM 11729	37°C, BHI
*Enterococcus faecium*	ATCC 19434	37°C, MRS

### 2.2. Antimicrobial activity analysis

The antimicrobial activity of the isolates was determined using the agar well diffusion assay, as described by Jozala et al. (2005). Briefly, the isolates were cultured in MRS medium for 15 h and adjusted the optical density as 1.0. The supernatant fractions were obtained by centrifugation (10,000 × *g* for 10 min) and filtration through a 0.22 μm filter to obtain cell-free supernatants (CFS). The CFS was neutralized with 5 N NaOH. Subsequently, 100 μL of the filtered supernatants was added to 10 mm diameter wells created in a BHI agar plate previously spread with overnight-cultured *Listeria monocytogenes*. The plates were incubated at 37°C overnight, and the inhibitory activity was determined by measuring the size of the inhibition halo around the well, compared to ampicillin (1 mg/mL) as a control.

### 2.3. Molecular characterization of bacteriocin

The neutralized CFS was used for bacteriocin purification. In details, ammonium sulfate (Sigma-Aldrich) was gently added to the sample to obtain 60% saturation and stirred for 15 h at 4°C. Then, it was desalted by dialysis with a cellulose semipermeable membrane (Spectra/Por dialysis tube membrane, MW cut-off 1 kDa, no. 132638; Spectrum Labs, Irving, TX, United States) in 20 mM potassium phosphate buffer (pH 7.0). Then, bacteriocin was purified with anion exchange chromatography (DEAE cellulose; Sigma-Aldrich). To determine the molecular weight of the purified bacteriocin, tricine-sodium dodecyl sulfate-polyacrylamide gel electrophoresis (Tricine SDS-PAGE) and gel overlay assay against *L. monocytogenes* KCTC 3569 (10^6^ CFU/mL) were conducted. The active bacteriocin area was determined by visual observation after incubating overnight at 37°C and the titers of an antimicrobial peptide expressed as arbitrary units (AU; mL^−1^ of medium). To determine mass spectra of bacteriocin, autoflex maX MALDI-TOF/TOF (Bruker Daltonics, Bremen, Germany) was used in the linear positive mode. The purified bacteriocin sample was desalted with C18-ziptip. FlexControl 3.4 software (Bruker Daltonics) was applied to acquire and process the spectra. Sample was mixed with matrix solution [Super-DHB (2,5-dihydroxybenzoic acid: 2-hydroxy-5-methoxy benzoic acid = 9: 1 (w/w)) in 0.1% trifluoroacetic acid/cetonitrile (1:1, v/v)] directly on the MALDI target and vacuum drying. Charged ions were distinguished in the *m*/*z* range of 1,000–10,000 Da, and 500 shots were accumulated per one spectrum.

### 2.4. Safety assessment

The hemolytic activity of EFEL8600 was analyzed by cultivating the strain on BHI medium containing 7% horse blood (MB CELL, Seoul, Republic of Korea) at 37°C for 24 h (Ryu and Chang, 2013). Then, hemolytic zones were compared with that of *L. monocytogenes* used as a positive control. The bile acid deconjugating activity of the strains were determined by culturing the strains on MRS agar with 0.5% taurodeoxycholic acid (w/v) under anaerobic conditions at 37°C for 48 h. Deconjugation of taurodeoxycholic acid created a white opaque precipitate of deoxycholate in the surrounding area of colonies. The concentration of DL-lactate was determined using high-performance liquid chromatography (HPLC) with an Agilent 1260 Infinity HPLC system (Agilent Technologies, CA, United States). For the analysis, a Shodex ORpak CRX-853 column (8.0 × 50 mm, Showa Denko, Tokyo, Japan) coupled with a CRX-G column (Showa Denko) was used. The supernatant was collected and filtered using a 0.22 μm microfilter membrane (Whatman) after cultivation in MRS medium for 15 h and the filtrate was used for HPLC analysis. CuSO_4_ (1 mM) was used as an eluent with 1 mL/min of flow rate at room temperature. Hyaluronidase activity was measured by incubating EFEL8600 strain with hyaluronic acid (3 mg/mL) in 12.5 mM CaCl_2_ at 37°C for 40 min (Ölgen et al., 2007). After reaction, a coloring reagent, *p*-dimethyl amino benzaldehyde (*p*DMAB) in glacial acetic acid and hydrochloric acid (1.5 mL, 9:1, v/v), was added, resulting in a reddish-purple colored product detected at 585 nm. All safety assessments in this study were conducted following the guidelines primarily established by FAO/WHO (2002).

### 2.5. Antibiotic resistance

Antibiotic resistance of EFEL8600 was determined by minimum inhibitory concentration (MIC) based on the Clinical and Laboratory Standards Institute (CLSI) method CLSI (2019). The ten antibiotics ampicillin, vancomycin, gentamicin, kanamycin, streptomycin, erythromycin, clindamycin, tetracycline, chloramphenicol, tylosine were tested followed by EFSA (2018) guideline. Then pre-cultured EFEL8600 (final concentration 5 × 10^5^ CFU/mL) was inoculated into Mueller-Hinton broth (Sigma-Aldrich, St. Louis, MO, United States) (100 μL) containing each antibiotic range of 0.125 to 256 mg/L. The plate was cultured under anaerobic conditions at 37°C for 48 h. The minimum concentration at which the strain did not survive was determined as MIC by visual observation.

### 2.6. Gastrointestinal stability

Tolerance to artificial gastrointestinal conditions were investigated by culturing the strain in pH adjusted PBS with HCl (pH 3.0 and pH 2.5) or PBS solution containing 0.3% (w/v) bile salt (Sigma-Aldrich) (Conway et al., 1987). After 0, 90, and 180 min incubation at 37°C, survival rate was estimated by viable cell counting on MRS agar and compared with reference strains. The adhesion of EFEL8600 to intestinal epithelial cells was assessed using Caco-2 and HT-29 epithelial cell lines, obtained from the Korean Cell Line Bank (Seoul, Republic of Korea). The cells were cultured in DMEM (Dulbecco’s modified Eagle’s medium; Hyclone, UT, United States) supplemented with fetal bovine serum (FBS; Hyclone), 1% penicillin (10,000 U/mL), and 1% streptomycin (10 mg/mL). Caco-2 and HT-29 cells were seeded at a density of 4.7 × 10^5^ cells/well in 24-well tissue culture plates without antibiotics. Bacterial cells (1 × 10^8^ CFU/mL) were resuspended in DMEM without antibiotics and applied to the Caco-2 and HT-29 cell monolayers. After incubating for 2 h at 37°C in a 5% CO_2_ incubator, a detachment solution containing 0.1% Triton X-100 and 0.1% trypsin-EDTA (Sigma-Aldrich) was used to detach the cells for 15 min. The detached cells were then washed with PBS and cultured on MRS agar plates to enumerate the adherent bacteria. The adherent bacteria were compared with reference probiotics. The experiments were performed in triplicate.

### 2.7. Protective effects on H_2_O_2_-induced intestinal damages

To analyze the gut barrier function of EFEL8600, the intestinal permeability was measured in the epithelial barrier model using 12 well Transwell^®^ inserts (polyester membrane with 0.4 μm pore size, 12 mm diameter; Costar, Corning Life Science, Kennebunk, United States) at a density of 5 × 10^4^ cells per cm^2^ of Caco-2 cell (Miyauchi et al., 2012). Caco-2 cell was cultivated until the transepithelial/transendothelial electrical resistance (TEER) value reached 200 Ω·cm^2^ which regarded as confluent by changing the medium every 48 h using Millicell-ERS (Millipore, Burlington, MA, United States). Then, 100 μM of H_2_O_2_ was treated to the Caco-2 cells (5 × 10^7^ CFU/well) for 30 min and TEER value was analyzed every 30 min after sensitization. The results were expressed as % TEER compared with the initial TEER value at T_0_ (before the addition of H_2_O_2_) for each insert using the formula: TEER (Ω·cm^2^)/initial TEER (Ω·cm^2^) × 100 (%). In addition, 100 μg/mL of fluorescein isothiocyanate (FITC)-dextran was treated to upper chamber in the dark condition at room temperature for 4 h. Samples (100 μL) from the lower chamber of each well were moved to an opaque black 96-well plate. Then, intensity of fluorescence was measured by a fluorescence spectrometer (LS55, Perkin Elmer Instruments, Waltham, United States) at excitation (485 nm) and emission (535 nm) wavelengths. The experiments were conducted in triplicate.

### 2.8. Antioxidative activity

The antioxidant activity of EFEL8600 was assessed by 2,2-diphenyl-1-picrylhydrazyl (DPPH) scavenging ability (Das and Goyal, 2015) using the three fractions: intact cells, cell-free culture supernatants (CFS) and, cell-free extracts (CFE). DPPH solution was prepared as 0.4 mmol/L and concentration of cultured cells were adjusted as 5 × 10^8^ cells/mL. Intact cells were made by resuspending in 0.85% NaCl, and CFS was collected by centrifugation after the pH was adjusted to 7.0 with 1 M of NaOH. For CFE preparation, cells were broken by sonicator (VP-050 N; Taitec Corp., Saitama, Japan) for 10 min (pulse 5 s on/5 s off at 35% amplitude), and the cell debris was eliminated by centrifugation (10,000 × *g*, 30 min). Then, 100 μL of each sample or ethanol (negative control) was reacted with 100 μL of prepared DPPH solution at 37°C in the dark condition for 30 min. Then, absorbance of samples was assessed at 517 nm by microplate reader (BioTek, Winooski, United States). The experiments were conducted in triplicate.

### 2.9. Nitric oxide assay

The inhibitory activities of the EFEL8600 on nitric oxide (NO) production were analyzed using LPS-induced RAW 264.7 cells (Yu et al., 2019). For heat-kill cell, bacterial cells (5 × 10^8^ cells/mL) were exposed to a heat treatment at 95°C for 30 min, and cell lysates were prepared as described in manufacturing CFE. The murine macrophage cell line RAW 264.7 (2 × 10^5^ cells/well) was retained in DMEM supplemented with 10% FBS (Hyclone) containing 1% penicillin–streptomycin at 37°C in 5% CO_2_ incubator. The 1 μg/mL LPS was treated to RAW 264.7 to induce NO production and heat-killed cells or lysates (5 × 10^7^ cells/well) were treated sequentially. After 24 h incubation, Griess reagent (Sigma-Aldrich) was treated to supernatant obtained from the each well and reacted in dark at 25°C for 15 min. Every absorbance at 540 nm was measured by a microplate reader (Bio-Tek). The experiments were conducted in triplicate.

### 2.10. Biochemical characteristics

The carbohydrate utilization ability of the *E. faecium* EFEL8600 was assessed using the API CHL kit (BioMériux Co., Marcy-l’Étoile, France) following the manufacturer’s instructions. After cultivation, bacterial cells were harvested and resuspended in API 50CH medium. A 120 μL aliquot of the suspension was inoculated into a tube containing strips. Mineral oil was then added to cover the tube, and the setup was incubated at 37°C for 48 h. The fermentation pattern was observed and recorded. A positive result was recorded when the blue indicator in the medium changed to yellow, indicating carbohydrate utilization.

### 2.11. Whole genome sequencing

To perform whole genome analysis, the genomic DNA of *E. faecium* EFEL8600 was extracted from the 15 h cultured cells in MRS medium (Difco) at 37°C using a bacterial genomic DNA prep kit (Solgent, Daejeon, Republic of Korea) according to the manufacturer’s instructions. The prepared genomic DNA was kept at −20°C for future use. DNA libraries were generated with Nanopore for long reads using MinION kit (Barcoding kit, EXP-NDB114; Ligation sequencing kit, LSK-109; Oxford Nanopore Technologies, Oxford, United Kingdom) and with Illumina Hi-seq for short read using TruSeq Nano DNA kit (Illumina, CA, United States). The quality of the reads was checked using FastQC v0.11.9 (Andrews et al., 2010), and Trimmomatic v0.36 program (Bolger et al., 2014) was used for filtering quality and removing Illumina adapter sequences. Hybrid genome assembly was conducted using short reads (Illumina) data and long reads (MinION) in SPAdes v3.15.2 (Bankevich et al., 2012). Finally, the complete genome sequence of EFEL8600 was annotated using the Prokka (prokaryotic genome annotation v1.13; Tatusova et al., 2016). Average nucleotide identity (ANI) is a measurement of genomic similarity in nucleotide-level between the coding sequence of two genomes, and OrthoANI is an improved method that compares orthologous fragment pairs (Lee et al., 2016). Orthologous (Ortho) ANI was analyzed using CJ Bioscience’s OrthoANI Tool (OAT), available on the EzBioCloud server.

### 2.12. *In silico analysis* of safety and probiotic properties

Genes related to virulence factors and toxin genes in EFEL8600 were analyzed by virulence factor database (VFDB) at http://www.mgc.ac.cn/cgi-bin/VFs/v5/main.cgi (Liu et al., 2022). The genome file in FASTA format was uploaded, and the genus was specified as *Enterococcus* to investigate the virulence factors predominantly found within this genus. A stringent search with cut-off values at >80% identity and >60% coverage and a less stringent search with cut-off values at >60% similarity, >60% coverage, and *E*-values <1e−10 were employed to discover putative virulence genes. In addition, BlastKoala search tool in the Kyoto Encyclopedia of Genes and Genomes (KEGG) database (Version 90.1) at https://www.kegg.jp/blastkoala/ was used to determine virulence factors and antibiotics resistance genes (Oh et al., 2022). Their transferability was analyzed by PHASTER at http://phaster.ca/ (Arndt et al., 2019).

Genes associated with probiotic properties, including resistance to gastrointestinal conditions, temperature, osmotic stress, oxidation, as well as genes related to bacteriocin production, were identified in EFEL8600 by searching the NCBI database. The sequence similarity of these genes was then compared using BLASTP (Lebeer et al., 2008).The presence of genes encoding bacteriocin in the EFEL8600 genome was determined using the web-based bacteriocin genome mining tool, BAGEL4, available at http://bagel4.molgenrug.nl/index.php. BAGEL tool identifies bacteriocins and their related clusters (van Heel et al., 2018). In details, it detects all genes related to bacteriocin processing, regulation, modification, passage, and immunity protein commonly located in near the putative bacteriocin sequence.

## 3. Results

### 3.1. Phenotype analysis of *Enterococcus faecium* EFEL8600

#### 3.1.1. Anti-microbial activity

**Figure 1:**
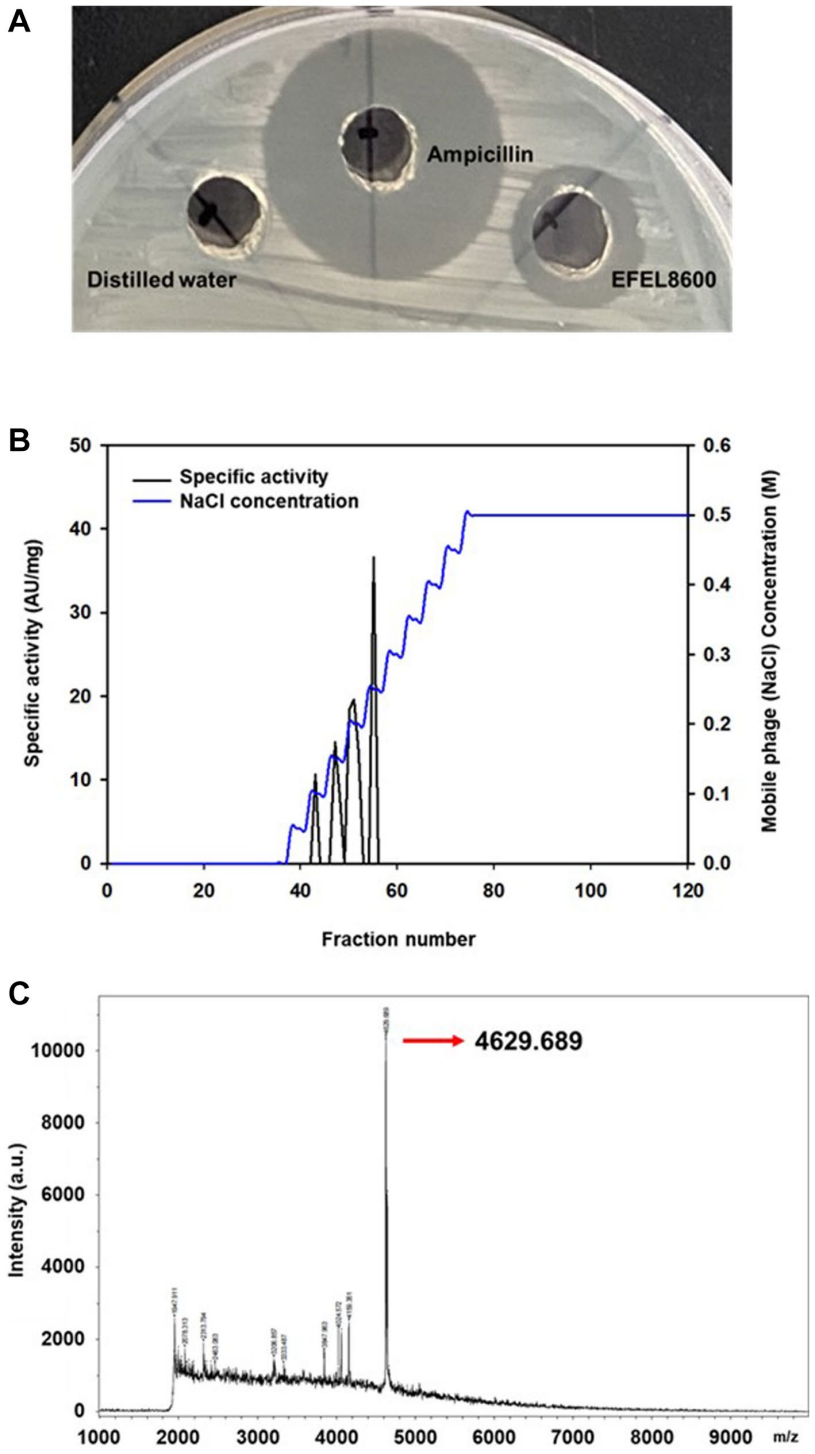
Antimicrobial activity of *Enterococcus faecium* EFEL8600 and purification of neutralized cell-free supernatant by anion exchange chromatography **(A)** Antimicrobial activity of neutralized cell-free supernatant from *E. faecium* EFEL8600 against *Listeria monocytogenes*. Positive control, ampicillin (1 mg/mL); negative control, distilled water. **(B)** The elution profiles of bacteriocin produced by *E. faecium* EFEL8600. Bacteriocin was eluted from the DEAE cellulose using a 11-step gradient with 0 to 0.5 M NaCl. The specific activity and corresponding mobile phage (NaCl) concentrations are indicated by the left and right y-axis, respectively. **(C)** MALDI-TOF/TOF spectrum of purified active bacteriocin sample (anion exchange chromatography).

To analyze the antimicrobial activity of *E. faecium* EFEL8600, the agar well diffusion assay was conducted by measuring the halo size around the well. As shown in Figure 1A, the neutralized cell-free supernatant from *E. faecium* EFEL8600 demonstrated a 4 mm clear inhibition zone and the positive control displayed a 10 mm clear zone of inhibition in the agar where *L. monocytogenes* was cultured as an indicator strain. Conversely, the negative control did not exhibit any observable zone of inhibition. This result revealed that the culture supernatant of EFEL8600 contained the antimicrobial compounds against *L. monocytogenes*.

#### 3.1.2. Molecular characterization of bacteriocin

Antimicrobial compounds of EFEL8600 strain were obtained by precipitating the culture supernatant using 60% ammonium sulfate, and the specific activities of the culture supernatant and precipitate fractions were measured as 1.28 and 36.98 AU/mg protein, respectively. This indicated a significant 28.89-fold increase in specific activity, revealing protein(bacteriocin)-like properties of the antimicrobial compounds. Subsequently, anion exchange chromatography (DEAE-cellulose) was employed to separate the active fraction, as shown in Figure 1B. Among the eluted fractions, fraction 55, obtained with 0.25 M NaCl, exhibited the highest activity against *L. monocytogenes*. This step led to a 28.74-fold increase in antimicrobial activity (Table 2). To determine the molecular weight of the purified bacteriocin, a gel overlay assay was performed (Supplementary Figure S1). Tricine SDS-PAGE analysis of the active fraction obtained from anion exchange chromatography revealed multiple peptide bands, while a distinct clear zone surrounding the 5 kDa region appeared upon incubation with *L. monocytogenes*. Comparing the marker proteins on the Tricine SDS-PAGE gel, the molecular weight of the bacteriocin was estimated to be approximately 4–5 kDa. To obtain an accurate measurement of the molecular weight, MALDI-TOF/TOF analysis was conducted on the 4–5 kDa peptide. As shown in Figure 1C, the mass spectrometry spectrum exhibited a sharp peak corresponding to a molecular weight of 4629.689 Da.

**Table 2:** Purification steps of bacteriocin produced by *Enterococcus faecium* EFEL8600.

Purification stage	Volume (mL)	Protein concentration (mg/mL)	Total activity (AU)	Specific activity (AU/mg)	Purification fold
Culture supernatant	1,000	7.8 ± 0.15^a^	10,000	1.28 ± 0.02	1.00 ± 0.00
Ammonium sulfate precipitation	30	4.32 ± 0.14	4,800	37.03 ± 1.25	28.89 ± 0.79
Anion exchange chromatography	5	0.55 ± 0.05	100	36.82 ± 3.06	28.74 ± 2.4

a Data represents means ± standard deviation (SD) from triplicate determinations.

#### 3.1.3. Safety assessment

**Figure 2:**
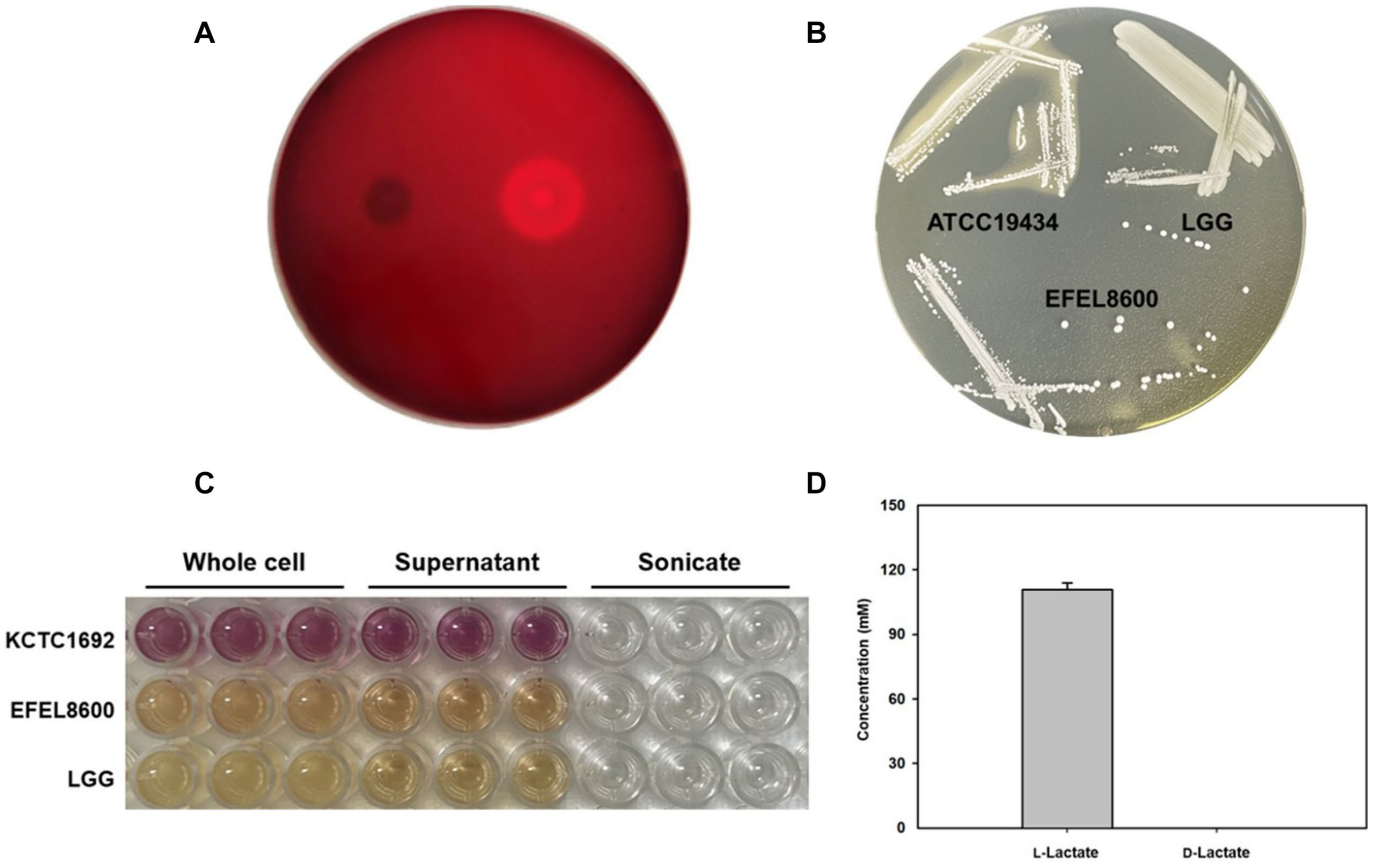
Safety analysis of *Enterococcus faecium* EFEL8600 *in vitro*. **(A)** Hemolytic activity analysis of *E. faecium* EFEL8600. Hemolytic activity was measured on BHI agar medium containing 7% horse blood. Right, positive control, *Listeria monocytogenes*, showing clear zone around the cell drop; left, the EFEL8600 strain. **(B)** Bile salt hydrolase activity of *E. faecium* EFEL8600. Plates were incubated anaerobically for 48 h at 37°C. ATCC 19434, *Enterococcus faecium* ATCC 19434 used as positive control; LGG, *Lacticaseibacillus* GG used as a negative control. **(C)** Hyaluronidase activity by optical density at 585 nm. Hyaluronidase enzyme is responsible for the degradation of hyaluronic acid, resulting in the production of N-acetylglucosamine which reacts with the DMAB reagent, causing a color change from yellow to purple. KCTC 1692, *Staphylococcus aureus* KCTC 1692. **(D)** DL-lactate level in the supernatant of the *E. faecium* EFEL8600 strain. Results are expressed as means ± SD (*n* = 3).

Safety evaluation of EFEL8600 for food use involved the analysis of hemolytic activity, bile salt deconjugation activity, hyaluronidase activity, antibiotic resistance, and D- and L-lactate production. Hemolytic activity was assessed on 7% horse blood agar, and no clear zones were observed around EFEL8600 colonies, while a clear zone was observed for *L. monocytogenes* (Figure 2A). Bile salt deconjugation activity was analyzed on MRS agar supplemented with 0.5% taurodeoxycholic acid. EFEL8600 and LGG showed no deconjugation activity, while *E. faecium* ATCC 1943 (positive control) resulted in a white precipitate of deoxycholate (Figure 2B). Hyaluronidase activity was determined by incubating whole cells, supernatant, and lysate fractions of EFEL8600 with hyaluronic acid. EFEL8600 and LGG showed no color changes, whereas *Staphylococcus aureus* KCTC 1692 (positive control) exhibited purple color, indicating hyaluronidase activity (Figure 2C). The D- and L-lactate production profile of EFEL8600 was analyzed by measuring lactate concentrations in the culture medium. Only L-lactate was detected at a concentration of 110 mM, indicating exclusive production of L-lactate without synthesis of the D-form stereoisomer (Figure 2D). To examine antibiotic resistance, a minimum inhibitory concentration (MIC) test was conducted following EFSA guidelines (Table 3). Among the nine tested antibiotics, EFEL8600 exhibited MIC levels within the cut-off values for nine antibiotics. However, erythromycin exceeded the cut-off value, showing a MIC of 128 μg/mL.

**Table 3:** Antibiotic resistance of *Enterococcus faecium* EFEL8600.

Antimicrobial agent	MIC^a^ (μg/mL)	EFSA cut off^b^ (μg/mL)
Ampicillin	0.5	2
Vancomycin	0.25	4
Gentamicin	32	32
Kanamycin	512	1,024
Streptomycin	64	128
Erythromycin	128	4
Clindamycin	2	4
Tetracycline	0.5	4
Chloramphenicol	4	16
Tylosine	4	4

a MIC, minimum inhibitory concentration.

b Microbiological cut-off values of *Enterococcus faecium* according to EFSA (2018).

Based on the results, EFEL8600 demonstrated no significant hemolytic activity, bile salt deconjugation activity, hyaluronidase activity, or D-lactate production, indicating its safety for food use. However, the observed resistance to erythromycin raises concerns regarding the potential transferability of resistance genes to other members of the intestinal microbiome. Given that mobile elements such as plasmids or phages are the main vehicles for gene transfer in lactic acid bacteria, genetic analysis is required to determine whether the antibiotic resistance genes in EFEL8600 are intrinsic or acquired. The details of this analysis will be discussed in the subsequent section of this study.

#### 3.1.4. Stability under low pH and bile salt and cell adhesion ability

**Figure 3:**
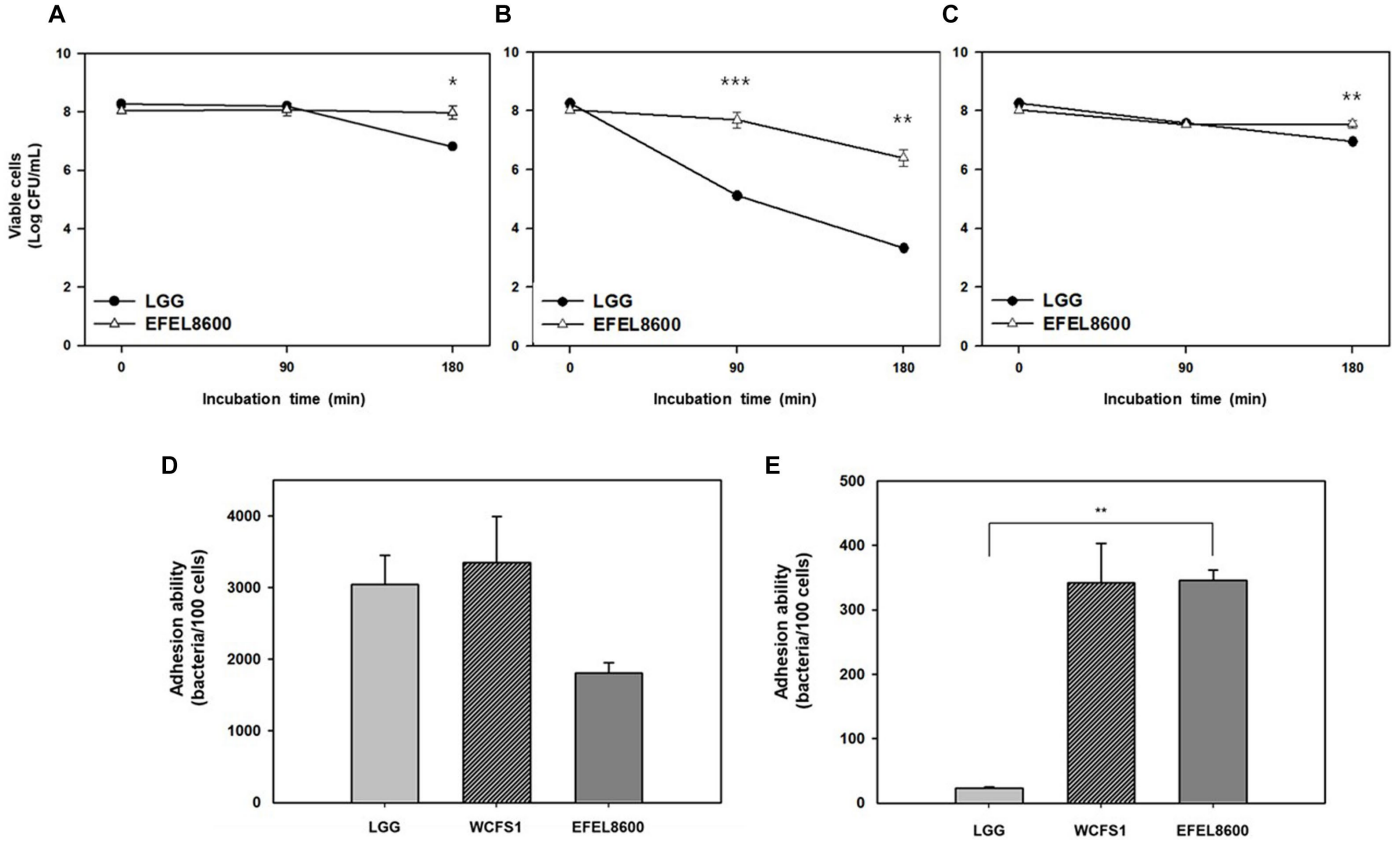
Intestinal stability and adhesion ability of *Enterococcus faecium* EFEL8600. Viability of *E. faecium* EFEL8600 under various simulated gastrointestinal condition **(A)** pH 3.0, **(B)** pH 2.5, and **(C)** 0.3% bile salt. Significant differences compared with *Lacticaseibacillus rhamnosus* GG (LGG) are represented with asterisks (^*^p < 0.05, ^**^p < 0.01, ^***^p < 0.001). Intestinal adhesion ability of *E. faecium* EFEL8600 against **(D)** Caco-2 cells, and **(E)** HT-29 cells as colonic epithelial cells. Results are expressed as means ± SD (*n* = 3).

The stability of EFEL8600 under gastrointestinal conditions was examined by measuring its tolerance to acid and bile salts (Figures 3A–C). The EFEL8600 strain demonstrated significantly higher resistance at pH 3.0 (7.9 log CFU/mL) and pH 2.5 (6.4 log CFU/mL) after a 180 min compared to the reference probiotic LGG. Moreover, when incubated in a 0.3% bile salt solution for 180 min, the EFEL8600 strain exhibited greater cell survival (7.6 log CFU/mL) compared to LGG (7.0 log CFU/mL) (Figure 3C). This result showed that EFEL8600 possesses high viability under simulated gastrointestinal conditions, suggesting its potential to reach the large intestine in a viable state. Subsequently, the ability of EFEL8600 to adhere to intestinal epithelial cells was analyzed by incubating the strain on a Caco-2 and HT-29 cell monolayer (Figures 3D,E). The result revealed that a total of 1,808 ± 142 cells adhered to 100 Caco-2 cells, and 345 ± 16 cells adhered to 100 HT-29 cells. Notably, the adhesion ability of EFEL8600 was comparable to that of WCFS1 in HT29 cells. This result indicated that EFEL8600 exhibits proficient adhesion capacity similar to that of a commercially available probiotic strain.

#### 3.1.5. Protective activity on intestinal barrier

**Figure 4:**
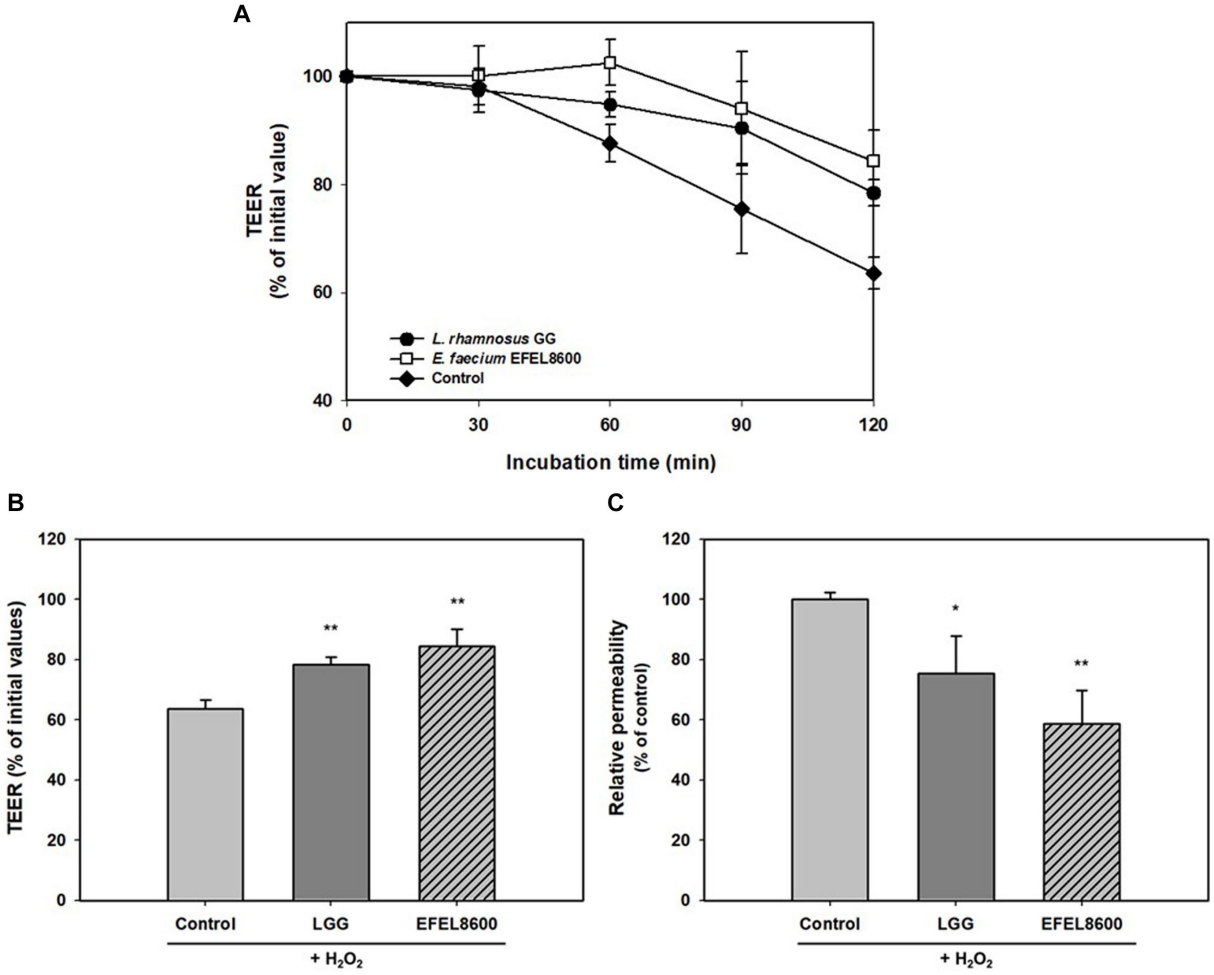
Protective effect of *Enterococcus faecium* EFEL8600 on intestinal barrier function. Caco-2 cell monolayers were pretreated with bacterial strain for 30 min, and the monolayers were exposed to H_2_O_2_ (1 mM). TEER was measured every 30 min **(A)** until 2 h **(B)** after H_2_O_2_ treatment. FITC-dextran fluorescence intensity **(C)** was measured at 4 h later after TEER measurement and expressed in % compared to control value. *Lacticaseibacillus rhamnosus* GG (LGG) was used as a positive control (^*^p < 0.05, ^**^p < 0.01). All data are expressed as mean ± SD (*n* = 3).

The protective effect of EFEL8600 on gut barrier function was assessed by measuring permeability with TEER values and the fluorescence level of FITC dextran using H_2_O_2_-induced confluent Caco-2 cell monolayers (Figure 4). The addition of H_2_O_2_ to Caco-2 cell monolayers led to a decrease in TEER by 63.6% after 120 min, indicating increased monolayer permeability. However, treatment with EFEL8600 resulted in a higher TEER value of 84.4%, compared to the positive control LGG (78.4%) (Figures 4A,B). This result showed the protective effect of EFEL8600 against H_2_O_2_-induced epithelial cell damage. Similarly, when the permeability of Caco-2 cells was assessed using FITC-dextran, EFEL8600 exhibited lower permeability (58.6%) than LGG (75.3%) and the control group (100%, *p* < 0.01) (Figure 4C). Collectively, these results indicated that treatment with EFEL8600 has a defensive effect on gut barrier function by protecting the epithelial membrane against H_2_O_2_-induced oxidative damage.

#### 3.1.6. Antioxidative and anti-inflammatory activities

**Figure 5:**
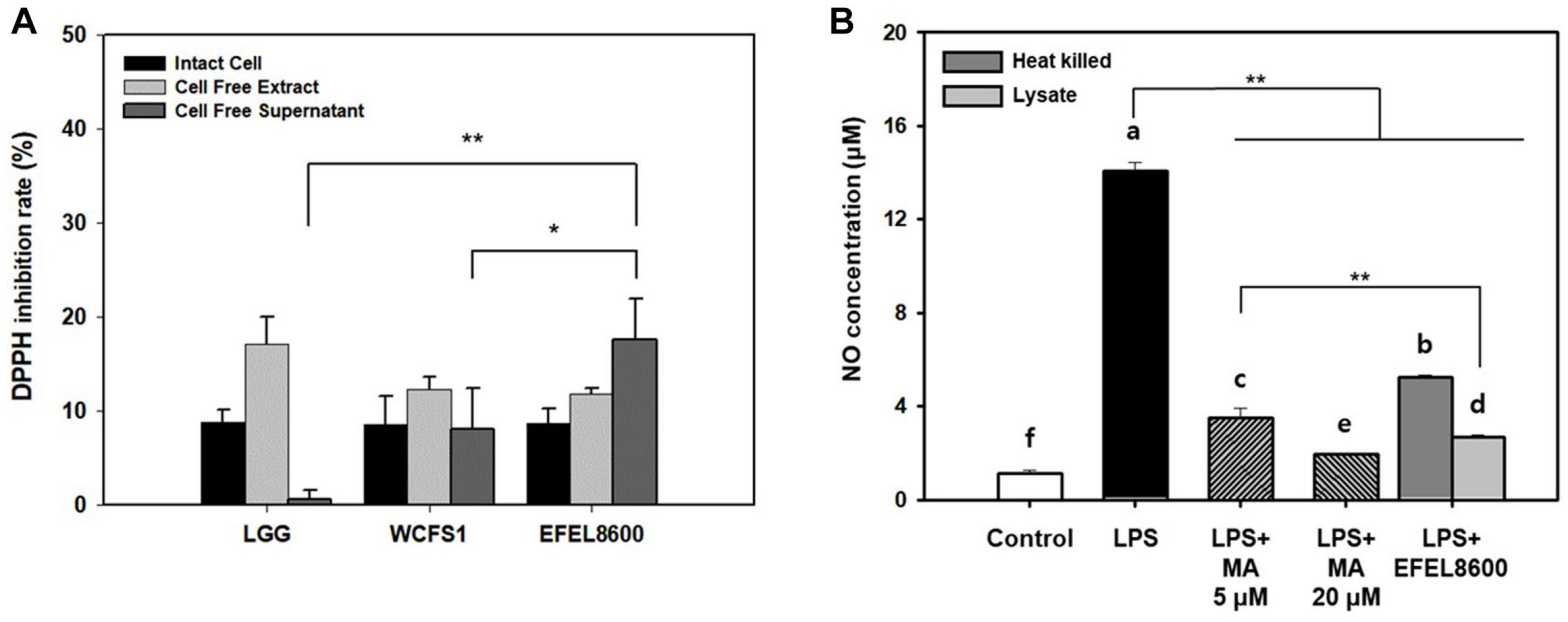
Antioxidant and anti-inflammatory activity of *Enterococcus faecium* EFEL8600. **(A)** Antioxidant activity of *E. faecium* EFEL8600 measured by the DPPH inhibition assay. Fractions of intact cells, cell-free extract, and cell-free culture supernatant were used. **(B)** Anti-inflammatory activity of *E. faecium* EFEL8600 in LPS-induced RAW 264.7 cells. Asterisks on the error bars indicate significant differences, and one-way analysis of variance (ANOVA) with Tukey’s method was used to analyze differences between multiple groups (^*^p < 0.05, ^**^p < 0.01). All data are expressed as mean ± SD (*n* = 3).

The antioxidative activity of EFEL8600 was evaluated by measuring its DPPH scavenging ability using intact cells, cell-free extract (CFE), and cell-free supernatant (CFS) (Figure 5A). The three fractions of EFEL8600 exhibited higher DPPH scavenging ability, with the highest value observed in CFS (17.5%), followed by CFE (11.7%) and intact cells (8.7%), compared to the positive controls, LGG (0.6%) and WCFS1 (8.0%). This result indicated that EFEL8600 possesses a protective effect against oxidative stress in the human intestine. Furthermore, the anti-inflammatory effect of EFEL8600 was assessed by analyzing the inhibitory activities of heat-killed cells or cell lysate on NO production in LPS-induced RAW 264.7 cells (Figure 5B). The result demonstrated that both heat-killed cells and cell lysate significantly suppressed NO production, with values of 5.2 μM and 2.7 μM, respectively, at corresponding levels of 5 μM and 20 μM of methyl arginine, a NO synthase inhibitor. These findings indicated that EFEL8600 possesses superior antioxidative and anti-inflammatory activities.

#### 3.1.7. Biochemical characterization

The biochemical characteristics of *E. faecium* EFEL8600 were compared to the type strain, *E. faecium* ATCC 19434. As shown in Supplementary Table S1, both strains shared the ability to metabolize L-arabinose, ribose, D-galactose, glucose, fructose, mannose, mannitol, mannopyranoside, N-acetyl-glucosamine, arbutin, esculin, salicin, cellobiose, maltose, lactose, melibiose, sucrose, trehalose, and gentiobiose. However, EFEL8600 exhibited additional metabolic capabilities, including the utilization of glycerol, raffinose, and starch, which were not observed in the type strain. This finding highlights the unique carbohydrate metabolic pathways present in EFEL8600. EFEL8600 has been deposited in the Korean Collection for Type Cultures (KCTC) under accession no. KCTC 14743BP.

### 3.2. Genome-based analysis *Enterococcus faecium* EFEL 8600

#### 3.2.1. Whole genome sequence analysis

**Figure 6:**
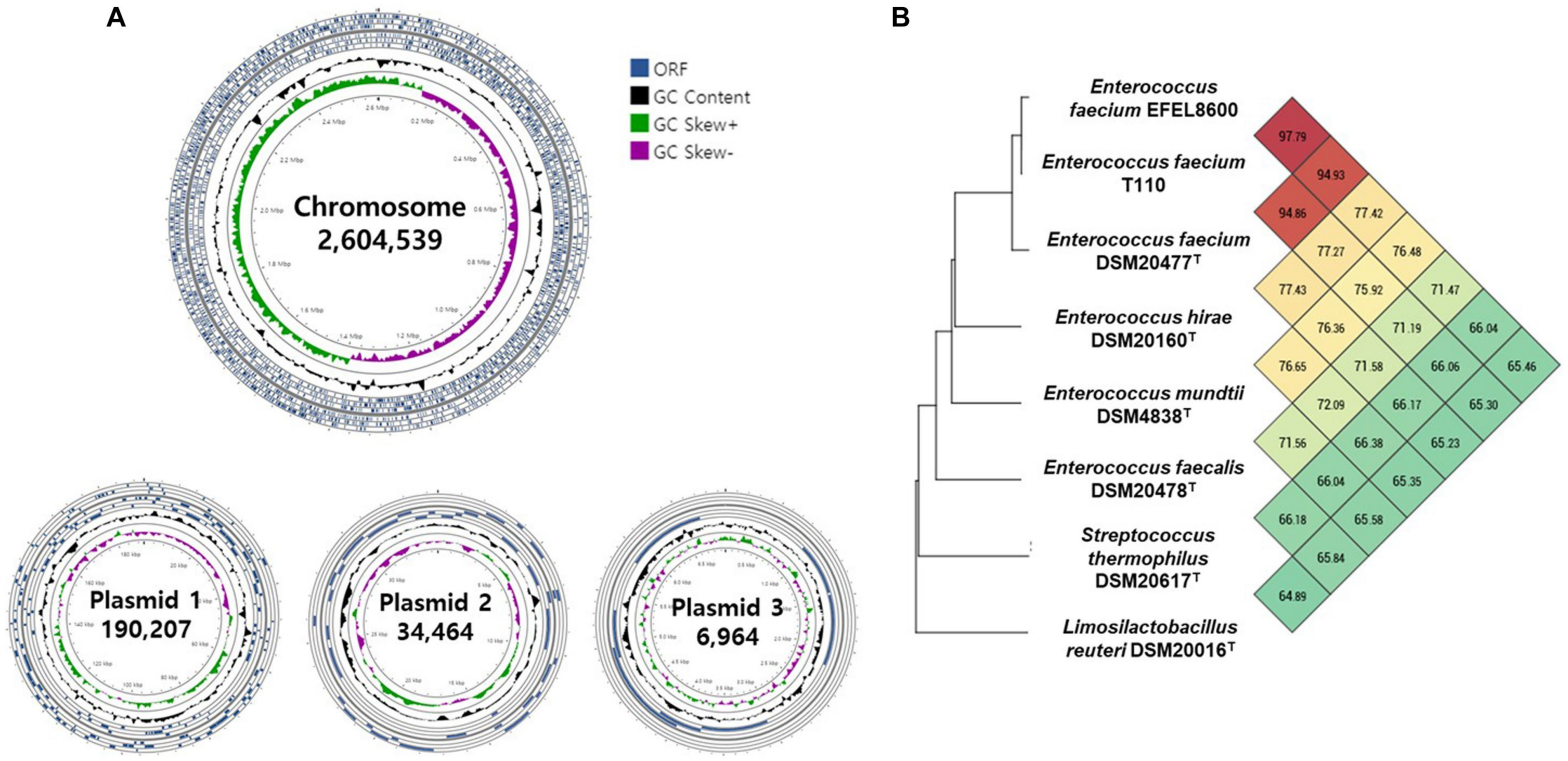
Graphical genome maps and OrthoANI analysis of *Enterococcus faecium* EFEL8600 compared with other lactic acid bacteria. **(A)** Graphical maps of the *E. faecium* EFEL8600 chromosome and plasmids created by the CG View Server. **(B)** Heat map of OrthoANI of *E. faecium* EFEL8600 compared with other lactic acid bacteria. Each cell represents the OrthoANI values between the row and the corresponding genomes of the column. The phylogenetic tree on the left represents a phylogenetic distance constructed by CJ Bioscience’s OAT, available on the EzBioCloud server.

The genetic characteristics of EFEL8600 were analyzed through the sequencing of its genomic DNA using the Nanopore and Illumina platforms. Nanopore sequencing generated 118,536 high-quality reads, resulting in a total of 1,518,801,768 base pairs (bp) with an average read length of 12,813 bp. Illumina sequencing produced a total of 14,865,056 high-quality reads, yielding 2,244,623,456 bp with an average read length of 151 bp. The hybrid genome assembly process (Figure 6A) led to the generation of a circular chromosome measuring 2,604,539 bp with a G + C content of 38.47%. Additionally, three plasmids were identified with sizes of 190,207 bp (G + C content: 35.10%), 34,464 bp (G + C content: 34.99%), and 6,964 bp (G + C content: 33.66%). The genomic features of EFEL8600, including the presence of 2,750 protein-coding genes, 69 tRNA genes, 18 rRNA genes, and 1 ncRNA, are summarized in Supplementary Table S2. No CRISPR sequence was detected. To determine the taxonomy of EFEL8600, a comparative analysis was conducted by calculating its OrthoANI value with other Enterococci and lactic acid bacteria (Figure 6B). The result indicated that EFEL8600 exhibited OrthoANI values of 94.93 and 97.79% with *E. faecium* ATCC 19433 (DSM 20477) and *E. faecium* T110, respectively. It is worth noting that ANI values above 95%–96% are typically considered indicative of the same species (Yoon et al., 2017). Therefore, based on this genomic comparison, it is evident that EFEL8600 belongs to the species *E. faecium*.

#### 3.2.2. Safety-related genes

To investigate the presence of virulence factors in the genome of EFEL8600, two databases, the virulence factor database (VFDB) and BlastKOALA, were utilized. A total of 12 genes associated with virulence factors or undesirable metabolites were identified (Table 4). In VFDB, with *Enterococcus* specified as the genus, genes associated with adherence factors (*acm*, *cna*, and *sagA*), capsulation (*cpsA* and *cpsB*), biofilm formation (*bopD*), and hyaluronidase (*hyals*) were identified. These genes are commonly found in *Enterococcus* regardless of whether they are pathogenic or non-pathogenic (Natarajan and Parani, 2014). The genes related to adherence factors are often found as pseudo genes in *Enterococcus* due to insertion of phage sequence or deletion of nucleotide (Freitas et al., 2018). However, considering that there were no insertions or deletions in the amino acid sequence of these genes, and no phage sequences were identified, it is estimated that these genes are likely to exist in an intact form. It is worth noting that certain factors contributing to enterococcal virulence, such as adherence-related genes and biofilm formation, are advantageous in probiotic strains as they facilitate effective gut colonization, enhance adherence to intestinal walls, modulate the immune system indirectly, and provide protection against harmful bacteria (Krawczyk et al., 2021). Concerning capsule production-related genes, only seven genes (*cpsC*, *cpsD*, *cpsE*, *cpsG*, *cpsI*, *cpsJ*, and *cpsK*) are essential, and *cpsA* or *cpsB* do not produce capsules (Penas et al., 2013). While the *bopD* gene associated with biofilm formation is present, its expression could potentially be hindered due to the absence of the fsrABC operon, a regulator of its transcriptional activity (Shridhar et al., 2022). Regarding the hyaluronidase gene, despite its presence in the chromosomal DNA, no enzymatic activity was detected in the enzyme activity assay (Figure 2C). The Virulence Factor Database (VFDB) offers comprehensive information and analytical platform on bacterial virulence factors, initially compiled from experimentally validated sources, and augmented with genetic data from GenBank using Perl scripts (Chen et al., 2005). For this reason, while the prevalence of annotation errors may not be significant, their potential occurrence is influenced by various factors such as inaccuracies in data, evolutionary divergence, homology with non-virulence elements, and insufficient experimental validation. Therefore, depending on necessity, further validation through experimentation may be demanded for a more accurate verification. In BlastKOALA, genes associated with bacterial toxins, pore-forming toxins, and tyramine production were identified. However, despite the presence of pore-forming genes in the chromosomal DNA of EFEL8600, no hemolytic activity was observed *in vitro* (Figure 2A). These results indicated none-expression of virulence-related genes or defects in the catalytic action of the corresponding proteins. Regarding antibiotic resistance genes, a search was conducted in the genome of EFEL8600 using BlastKOALA (Table 5). A total of ten genes associated with antibiotic resistance were detected in the chromosome. To assess their transferability to other microorganisms in the human intestine, their presence in plasmid or phage-derived regions within the chromosome was analyzed (Supplementary Table S3). Analysis using PHASTER revealed the presence of a total of 9 phage regions (five in the chromosome and four in plasmid 1). Fortunately, the antibiotic resistance genes were not located within these phage sequences, suggesting a low likelihood of transfer to other bacteria. In summary, even though EFEL8600 contains some genes that may pose potential risks, it does not appear to have any highly severe genes that would negatively impact human health.

**Table 4:** List of virulence and undesirable genes detected in the genome of *Enterococcus faecium* EFEL8600 analyzed by database.

Database	Virulence factor class	Virulence factors^a^	Related genes^b^	Location
Virulence factor database (VFDB)	Adherence	Collagen adhesin precursor	*acm*	Chromosome
		Collagen binding protein	*cna*	Chromosome
		Fibronectin-binding protein	*sagA*	Chromosome
	Antiphagocytosis	Capsule	*cpsA/uppS*	Chromosome
		Capsule	*cpsB/uppS*	Chromosome
	Biofilm formation	BopD	*bopD*	Chromosome
	Enzyme	Hyaluronidase	undetermined	Chromosome
		Hyaluronidase	undetermined	Chromosome
BlastKOALA	Signaling and cellular processes	Exfoliative toxin A/B	*eta*	Chromosome
		Magnesium and cobalt exporter	*tlyC*	Chromosome
		Hemolysin III	*hlyIII*	Chromosome
	Amino acid metabolism	Tyrosine decarboxylase	*tdc-1*	Chromosome

a Virulence factors include toxins, adhesion proteins, and pathogenic hydrolases that allow microorganisms to settle in a particular host and cause disease.

b Name of virulence factor related genes.

**Table 5:** List of antimicrobial resistance gene analyzed by BlastKOALA and their locations in the genome of *Enterococcus faecium* EFEL8600.

Resistance	KEGG ID	Gene name	Protein ID	Region position	Location
Tetracycline resistance genes	K08151	MFS transporter, tetracycline resistance protein	HELCIOKC_00202	197,502–198,689	Chromosome
Macrolide resistance genes	K18231	Macrolide transport system ATP-binding/permease protein	HELCIOKC_01289	1,279,171–1,280,649	
	K19350	Lincosamide and streptogramin A transport system	HELCIOKC_01980	2,015,439–2,016,941	
	K08217	MFS transporter, DHA3 family, macrolide efflux protein	HELCIOKC_01888	1,917,404–1,918,681	
Vancomycin resistance	K07260	Zinc D-Ala-D-Ala carboxypeptidase	HELCIOKC_00336	334,559–335,383	
Cationic antimicrobial peptide (CAMP) resistance, dltABCD operon	K03367	D-alanine-poly(phosphoribitol) ligase subunit 1	HELCIOKC_00775	747,295–748,809	
	K03739	Membrane protein involved in D-alanine export	HELCIOKC_00774	746,093–747,298	
	K14188	D-alanine--poly(phosphoribitol) ligase subunit 2	HELCIOKC_00773	745,815–746,048	
	K03740	D-alanine transfer protein	HELCIOKC_00772	744,547–745,812	
Cationic antimicrobial peptide (CAMP) resistance, lysyl-phosphatidylglycerol (L-PG) synthase MprF	K14205	Phosphatidylglycerol lysyltransferase	HELCIOKC_00441	441,266–443,866	
			HELCIOKC_01260	1,248,947–1,251,520	

#### 3.2.3. Probiotic-related genes

To investigate the probiotic properties of EFEL8600 at the genomic level, genome annotation was performed using Prokka v 1.14. As shown in Table 6, genes associated with probiotic traits were identified, which are associated with acid or bile salt tolerance, adhesion to epithelial cells, stress scavenging, and bacteriocin production. Specifically, the EFEL8600 genome retained F0F1 ATP synthase subunits and cation antiporters, which are involved in low pH tolerance. These genes contribute to the regulation of cytoplasmic pH by hydrolyzing ATP to pump H^+^ ions out of cells, maintaining pH homeostasis (Soni et al., 2022). These genes are commonly found in other *Enterococcus* spp. that contribute to their stress resistance, especially acid tolerance (Kopit et al., 2014). Moreover, choloylglycine hydrolase and 3-dehydro-bile acid delta(4,6)-reductase were identified as genes related to bile salt tolerance in EFEL8600 (Kim et al., 2020). In response to temperature stress, several cold shock proteins and chaperone proteins were detected. Additionally, the potassium/sodium uptake protein NtpJ was found to be involved in osmotic stress response (Checchetto et al., 2016). Furthermore, several antioxidant-related genes, including glutathione amide reductase, NADH oxidase, NADH dehydrogenase-like protein, and thioredoxin reductase, were identified. These genes play a role in interrupting chain reactions by eliminating intermediate free radicals and preventing oxidation by neutralizing reactive oxygen species (Mishra et al., 2015). In addition, these genes represent mechanisms frequently found in *Enterococcus* to resist oxidative stress conditions (Zhao et al., 2010). Finally, a bacteriocin-related gene, enterocin P, was found on plasmid 1 of EFEL8600.

**Table 6:** Genes related with probiotic characteristics in genome of *Enterococcus faecium* EFEL8600 searched in NCBI database.

Categories	Related protein	Protein ID	Location
pH	Alkaline phosphatase synthesis sensor protein PhoR	HELCIOKC_02258	2297771.2298475
	Alkaline phosphatase synthesis transcriptional regulatory protein PhoP	HELCIOKC_02259	2297771.2298475
	ATP synthase subunit a	HELCIOKC_02115	2152928.2153647
	ATP synthase subunit c	HELCIOKC_02116	2153700.2153915
	ATP synthase subunit b	HELCIOKC_02117	2154006.2154530
	ATP synthase subunit delta	HELCIOKC_02118	2154517.2155059
	ATP synthase subunit alpha	HELCIOKC_02119	2155082.2156638
	ATP synthase gamma chain	HELCIOKC_02120	2156652.2157554
	ATP synthase subunit beta	HELCIOKC_02121	2157577.2158983
	ATP synthase epsilon chain	HELCIOKC_02122	2158997.2159419
	Na(+)/H(+) antiporter	HELCIOKC_00070	60873.62024
	Sodium, potassium, lithium and rubidium/H(+) antiporter	HELCIOKC_00405	403381.405459
	Cadmium, cobalt and zinc/H(+)-K(+) antiporter	HELCIOKC_00602	599184.600083
	Cadmium, zinc and cobalt-transporting ATPase	HELCIOKC_02240	2273209.2275293
Bile	Choloylglycine hydrolase	HELCIOKC_02395	2396881.2397855
	3-dehydro-bile acid delta(4,6)-reductase	HELCIOKC_02378	2385385.2386638
Temperature	Cold shock-like protein CspLA	HELCIOKC_00004	2163.2360
		HELCIOKC_00836	805838.806038
		HELCIOKC_02232	2263849.2264049
	Cold shock protein 1	HELCIOKC_00025	20750.20950
	Cold shock-like protein	HELCIOKC_00178	170417.170647
	Cold shock protein CspD	HELCIOKC_00599	597639.597839
	Copper chaperone CopZ	HELCIOKC_01658	1666024.1666251
	Chaperone protein DnaJ	HELCIOKC_01817	1839293.1840060
	Copper chaperone CopZ	HELCIOKC_02244	2278245.2278454
	Chaperone protein DnaK	HELCIOKC_02482	2481359.2483188
	Chaperone protein DnaJ	HELCIOKC_02483	2483339.2484505
	Chaperone protein ClpB	HELCIOKC_02581	2596575.2599184
	60 kDa chaperonin	HELCIOKC_01006	987838.989463
Osmotic stress	Potassium/sodium uptake protein NtpJ	HELCIOKC_02084	2121415.2122770
Oxidation	Glutathione amide reductase	HELCIOKC_00360	357813.359135
	Glutathione reductase	HELCIOKC_01351	1355702.1357048
	Glutathione transport system permease protein	HELCIOKC_01484	1481033.1481995
	Glutathione biosynthesis bifunctional protein	HELCIOKC_01491	1491394.1493661
	Glutathione peroxidase	HELCIOKC_01812	1834603.1834713
	Glutathione-regulated potassium-efflux system ancillary protein KefG	HELCIOKC_01836	1860969.1861646
	NADH oxidase	HELCIOKC_01719	1727534.1728829
	NADH peroxidase	HELCIOKC_01828	1851520.1852899
	NADH oxidase	HELCIOKC_02058	2090585.2091949
	NADH dehydrogenase	HELCIOKC_00073	63382.64911
	Thioredoxin-like protein YtpP	HELCIOKC_01623	1631077.1631397
	Thioredoxin	HELCIOKC_02069	2104621.2104935
	Thioredoxin reductase	HELCIOKC_02179	2214907.2215833
	Thioredoxin-like protein YtpP	HELCIOKC_01623	1631077.1631397
	Quinone oxidoreductase 2	HELCIOKC_01296	1290560.1291411
	Quinone reductase	HELCIOKC_02266	2303892.2304446
	Hydroperoxide reductase C	HELCIOKC_00074	64921.65484
Bacteriocin	Bacteriocin enterocin-P	OEFIBKHD_00190	181054.181269

#### 3.2.4. Bacteriocin-producing genes

To identify genes associated with bacteriocin production, BAGEL 4 and SignalP-6.0 were used to investigate detailed gene clusters and signal peptide presence (Table 7). As a result, three bacteriocins were found on plasmid 1, namely Enterocin L50a, Enterocin P, and Enterolysin A, while one bacteriocin, Enterocin Q, was detected on plasmid 3. The genes and their clusters were summarized in Table 7. Enterocin L50a, Enterocin P, and Enterocin Q exhibited a 100% match with the bacteriocins derived from *E. faecium* listed in the BAGEL 4 database. In contrast, Enterolysin A displayed a relatively low match percentage of 35.93% with the bacteriocins listed in the database, and its specific origin remains unclear. In addition, the predicted sizes of Enterocin L50a, Enterocin P, Enterolysin A, and Enterocin Q were 4.84, 7.81, 44.11, and 3.74 kDa, respectively. Among them, only Enterocin P possessed a signal peptide for protein secretion, and its size, excluding the signal peptide, was 4.84 kDa. Interestingly, in this study, a molecular weight determination revealed the presence of an antimicrobial peptide with an observed size of 4.63 kDa (Supplementary Figure S1). This peptide is believed to correspond to either Enterocin L50a or Enterocin P, as they are among the four bacteriocin genes encoded in the EFEL8600 genome. The other bacteriocin genes were determined to have low expression levels or were not expressed.

**Table 7:** Bacteriocin related gene of *Enterococcus faecium* EFEL8600 predicted by BAGEL 4 (Bacteriocin Genomics Elimination 4).

Name	Orgin	Start	End	Match (%)	Sequence	Molecular weight (kDa)	Location
Enterocin L50a	*E. faecium*	167,506	167,640	100	MGAIAKLVAKFGWPIVKKYYKQIMQFIGEGWAINKIIEWIKKHI	4.84	Plasmid 1
Enterocin P	*E. faecium*	181,054	181,269	100	^a^MRKKLFSLALIGIFGLVVTNFGTKVDAATRSYGNGVYCNNSKCWVNWGEAKENIAGIVISGWASGLAGMGH	7.81	Plasmid 1
						^b^4.84	
Enterolysin A	Unclear	19,876	21,081	35.93	MENQNESLIKQYVKRRAKRRLFLWLFGTSAGLITILITVFVTLFLILAAGSIDNSDSDSSSGGEAFTGEYSEGLPIYKEIKGRGPFSDEIAQYAVGAAVKYKLLPSVILSQYGYESAFGTSASARNDLNYFGITWFDGCLFPKGTARGIGGIEGGWYMKFPNSKAAFSYYGFMVATQSNFNACVGNKSPGASLLILGRGGYAAAGITEDSPYYTGCMSIITSNKLTEYDEFAIKHWGEGGNDNGTITGEWTNPFPGSSLDKSSFSGGQLFGTNPGGEFRPNGFHDGLDFGSVDHPGSEIHAVHGGKVVYVGNPGISGLGACVIVINYDGLNMVYQEFANSTGNSRVKVGDQVKVGQVIGIRDTAHLHLGFARTDWRQAQGHAFTDDGTWIDPLPFLNSSKK	44.11	Plasmid 1
Enterocin Q	*E. faecium*	239	341	100	MNFLKNGIAKWMTGAELQAYKKKYGCLPWEKISC	3.74	Plasmid 3

a Underlined are the signal peptide sequences predicted by SignalP-6.0.

b Molecular weight (kDa) excluding signal peptide.

## 4. Discussion

Soymilk, a protein and mineral-rich liquid extract of soybean grains (Jinapong et al., 2008), is an affordable alternative to cow’s milk for individuals with lactose intolerance, milk protein allergy, or those who exclude milk from their diet (Yu et al., 2021). Fermentation of soymilk with lactic acid bacteria can enhance its sensory qualities by reducing undesirable beany flavors, producing organic acids and aromatic compounds (Kumari et al., 2022), and altering its rheological properties (Yang et al., 2021). Previous studies have shown that use of *Lactobacillus harbinensis* M1 increased the levels of aromatic compounds, 2,3-butanedione and acetoin and use of *L. fermentum* decreased the levels of off-flavors in soymilk (Zheng et al., 2020; Zhu et al., 2020). The use of lactic acid bacteria in fermentation can also improve the nutritional value of soymilk by increasing the bioavailability of minerals and indigestible oligosaccharides, such as raffinose and stachyose, through the action of β-glucosidase and α-galactosidase (Baú et al., 2015). Additionally, previous studies have reported the isoflavone bioconversion to aglycones and inhibition of angiotensin-converting enzyme (ACE) in fermented soymilk using *L. fermentum* and *L. casei* (Trupti et al., 2021). Nevertheless, soymilk is highly susceptible to *L. monocytogenes* contamination due to its nutrient-rich composition (Hsieh et al., 2011; Zhang et al., 2012). In this context, *E. faecium* EFEL8600 can be utilized as an effective starter in the fermentation of soymilk, aiming to prevent contamination by *L. monocytogenes*.

*E. faecium* is a prevalent species found in traditional Korean fermented soybean paste, meju (Jeong et al., 2019). This species possesses favorable characteristics for soybean fermentation, including rapid growth, flavor enhancement, and inhibition of pathogenic microorganisms in soybean cultures (Kumari et al., 2022). Additionally, *E. faecium* is equipped with several proteolytic enzymes capable of hydrolyzing soy proteins into small peptides or free amino acids (Moumita et al., 2018). However, due to its low resistance to salt, *E. faecium* is considered more suitable as a starter culture for soymilk rather than for high-salt soy products (>12% NaCl), such as doenjang (soybean paste) and ganjang (soybean sauce) (Martinez-Villaluenga et al., 2012).

Regarding *E. faecium* EFEL8600, it has demonstrated significant potential as a starter culture for fermented soymilk. Firstly, EFEL8600 exhibited rapid growth in soymilk, utilizing raffinose as a substrate, which is abundant in soybeans (Supplementary Table S1). Secondly, EFEL8600 does not raise major safety concerns, as it lacked hemolytic and bile salt deconjugation activities (Figures 2A,B) and showed a low risk of transferring antibiotic resistance genes (Table 5; Supplementary Table S3). Thirdly, EFEL8600 displayed notable protective effects on the intestinal barrier, as well as antioxidant and anti-inflammatory activities, while maintaining high gastrointestinal stability. Finally, EFEL8600 retains genes responsible for probiotic characteristics and bacteriocin production.

In recent years, the antibacterial activity of bacteriocins produced by enterococci, particularly *E. faecium* and *E. faecalis*, has been studied due to their potential use in controlling pathogenic bacteria such as *L. monocytogenes* (Fugaban et al., 2021). This advantageous characteristic has led to the utilization of various enterococcal strains in inhibiting the growth of foodborne pathogens during soymilk fermentation (Aspri et al., 2017). In our study, we found that the culture supernatant of EFEL8600 showed significant antibacterial activity against *L. monocytogenes* (Figure 1A). Further analysis of the antibacterial compound using mass spectrometry identified it as a bacteriocin with a molecular weight of 4.63 kDa, which is the similar molecular weight as enterocin L50a or enterocin P (Figure 1C). Based on the data, we have tried to determine the amino acid sequence of the bacteriocin by using MALDI-TOF-MS. However, this analysis presented a challenge as it was difficult to identify the correct amino acid sequence, probably due to the presence of remaining peptides. Instead, we predicted the sequence of bacteriocin based on genome sequence and sequence analysis tools. In cluster analysis, Enterocin L50a and Enterocin P, which are frequently detected in other *Enterococcus* species, were found to have secretion systems that corresponded to previous studies (Supplementary Table S4). In details, Enterocin L50a, the involvement of the ABC transporter system in the secretion of the bacteriocin from the producing cell has been reported (Teso-Pérez et al., 2021). This highlights the significance of ABC transporters in facilitating the export of Enterocin L50a across the cell membrane. In contrast, Enterocin P contains a signal peptide, typically located at the N-terminus of the protein (Martín et al., 2007). After synthesis, the signal peptide of Enterocin P is recognized, cleaved, and removed by specific enzymes, allowing it to be expelled with the protein during secretion, enabling its function outside the cell. No genes encoding transport proteins or signal peptides were detected for other bacteriocins such as Enterolysin A or Enterocin Q in this study. Based on the analysis of molecular weight and secretion mechanisms of bacteriocin, we predict that the 4.63 kDa bacteriocin corresponds to either Enterocin L50a or Enterocin P.

In conclusion, we demonstrated that *E. faecium* EFEL8600 is an antimicrobial probiotic that has health-promoting effects, such as improvement of gut barrier functions, antioxidant and anti-inflammatory activities. Therefore, *E. faecium* EFEL8600 can be applicable as probiotic starter for fermented soymilk with a health-promoting and antimicrobial safety function.

## Data availability statement

The original contributions presented in the study are included in the article/Supplementary material, further inquiries can be directed to the corresponding author.

## Author contributions

DHK: experiments/data collection, primary author (drafted the paper). S-AK: data analysis, primary author (drafted the paper). NGJ and J-HB: experiments/data collection. MTN and YMJ: data analysis. NSH: principal investigator (advisor, head of project, manager). All authors contributed to the article and approved the submitted version.

## Funding

This work was supported by Korea Institute of Planning and Evaluation for Technology in Food, Agriculture and Forestry (IPET) through High Value-added Food Technology Development Program, funded by Ministry of Agriculture, Food and Rural Affairs (MAFRA) (322009-04-1-SB010).

## Conflict of interest

The authors declare that the research was conducted in the absence of any commercial or financial relationships that could be construed as a potential conflict of interest.

## Publisher’s note

All claims expressed in this article are solely those of the authors and do not necessarily represent those of their affiliated organizations, or those of the publisher, the editors and the reviewers. Any product that may be evaluated in this article, or claim that may be made by its manufacturer, is not guaranteed or endorsed by the publisher.
