# Supplementary material for: Phenotypic and genomic analyses of bacteriocin-producing probiotic Enterococcus faecium EFEL8600 isolated from Korean soy-meju
Source: Front Microbiol. 2023 Sep 4;14:1237442. doi: 10.3389/fmicb.2023.1237442 (PMC10507247; doi:10.3389/fmicb.2023.1237442)
Supplement: Supplementary file 2 [file Data_Sheet_1.docx]

Supplementary Material

**Table S1 Carbohydrate utilization pattern of *Enterococcus faecium* EFEL8600 and DSM20477^Ta^**

| **Carbohydrate** | **EFEL8600** | **DSM20477^T^** |
| --- | --- | --- |
| Glycerol | W | - |
| Amygdalin | - | W |
| Raffinose | + | - |
| Starch | W | - |
| Tagatose | - | W |

^a^The results of *Enterococcus faecium* ATCC 19434 (DSM20477) were referenced (Ng et al., 2020). All strains were positive for L-arabinose, ribose, D-galactose, glucose, fructose, mannose, mannitol, mannopyranoside, N-acetyl-glucosamine, arbutin, esculin, salicin, cellobiose, maltose, lactose, melibiose, sucrose, trehalose, and gentiobiose (API 50 CHL). +, positive; -, negative; W, weakly positive

**Table S2 Genomic features of *Enterococcus faecium* EFEL8600**

| **Attribute** | **Total** | | **Chromosome** | | **Plasmid 1** | | **Plasmid 2** | | **Plasmid 3** | |
| --- | --- | --- | --- | --- | --- | --- | --- | --- | --- | --- |
|  | **Value** | **% of total** | **Value** | **% of total** | **Value** | **% of total** | **Value** | **% of total** | **Value** | **% of total** |
| Genomic size (bp) | 2,836,174 | 100.00% | 2,604,539 | 91.83% | 190,207 | 6.71% | 34,464 | 1.22% | 6,964 | 0.25% |
| DNA G+C (bp) | 1,083,128 | 38.19% | 1,001,967 | 38.47% | 66,757 | 35.10% | 12,060 | 34.99% | 2,344 | 33.66% |
| Total genes | 2,838 | 100.00% | 2,585 | 91.09% | 203 | 7.15% | 40 | 1.41% | 10 | 0.35% |
| CDS (coding) | 2,750 | 100.00% | 2,497 | 90.80% | 203 | 7.38% | 40 | 1.45% | 10 | 0.36% |
| tRNA genes | 69 | 100.00% | 69 | 100.00% | 0 | 0.00% | 0 | 0.00% | 0 | 0.00% |
| rRNA | 18 | 100.00% | 18 | 100.00% | 0 | 0.00% | 0 | 0.00% | 0 | 0.00% |
| ncRNA | 1 | 100.00% | 1 | 100.00% | 0 | 0.00% | 0 | 0.00% | 0 | 0.00% |

CDS, coding sequence; ncRNA, non-coding RNA; pseudogenes, nonfunctional segments of DNA; CRISPR sequence, clustered regularly interspaced short palindromic repeat sequences.

**Table S3 List of prophage regions in the genome of *Enterococcus faecium* EFEL8600 predicted by PHASTER**

| **Region** | | **Region length** | **Completeness** | **Score** | **Total proteins** | **Region position** | **Most common phage** | **GC (%)** |
| --- | --- | --- | --- | --- | --- | --- | --- | --- |
| Chromosome | 1 | 7.4 kb | Incomplete | 40 | 4 | 609,370-616,797 | PHAGE_Synech_ACG_2014f_NC_047712 | 33.49 |
|  | 2 | 42.9 kb | Incomplete | 40 | 6 | 639,794-682,734 | PHAGE_Lister_2389_NC_003291 | 35.94 |
|  | 3 | 7.4 kb | Incomplete | 20 | 6 | 1,611,874-1,619,319 | PHAGE_Bacill_vB_BtS_BMBtp14_NC_048640 | 36.93 |
|  | 4 | 41.4 kb | Intact | 110 | 51 | 2,333,631-2,375,089 | PHAGE_Entero_vB_IME197_NC_028671 | 35.29 |
|  | 5 | 9.5 kb | Incomplete | 20 | 7 | 2,389,602-2,399,123 | PHAGE_Lister_A006_NC_009815 | 31.42 |
| Plasmid 1 | 1 | 20.5 kb | Questionable | 80 | 13 | 46,107-66,656 | PHAGE_Escher_RCS47_NC_042128 | 35.30 |
|  | 2 | 22.3 kb | Incomplete | 50 | 8 | 101,409-123,806 | PHAGE_Lactob_phiAT3_NC_005893(2) | 34.59 |
|  | 3 | 29.3 kb | Questionable | 80 | 14 | 123,848-153,238 | PHAGE_Paenib_Xenia_NC_028837(2) | 34.23 |
|  | 4 | 8.1 kb | Incomplete | 10 | 3 | 154,748-162,946 | PHAGE_Lactob_phiAT3_NC_005893(2) | 35.04 |

**Supplement Figure 1.**

**
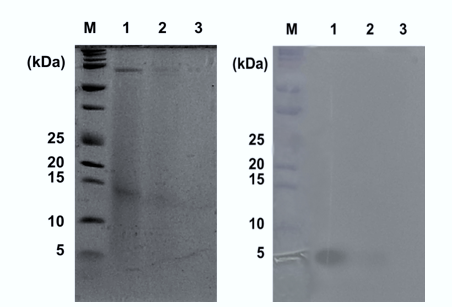
**

**Supplement Figure 1.** **Molecular mass of purified bacteriocin from *Enterococcus faecium* EFEL8600.** Left, Detection of inhibition zone related to bacteriocin activity. Lane M, molecular mass marker (2.0-250 kDa); lane 1 and 2, purified active bacteriocin sample (anion exchange chromatography); lane 3, negative control which has no protein. 16% separating gel of Tricine SDS-PAGE stained with 0.002% coomassie blue; Right, Tricine SDS-PAGE gel overlaid with soft agar inoculated with *Listeria monocytogenes*.
